# A Review of Recruitment, Adherence and Drop-Out Rates in Omega-3 Polyunsaturated Fatty Acid Supplementation Trials in Children and Adolescents

**DOI:** 10.3390/nu9050474

**Published:** 2017-05-10

**Authors:** Inge S. M. van der Wurff, Barbara J. Meyer, Renate H. M. de Groot

**Affiliations:** 1 Welten Institute, Research Centre for Learning, Teaching, and Technology, Open University of The Netherlands, 6419 AT Heerlen, The Netherlands; inge.vanderwurff@ou.nl; 2 School of Medicine, University of Wollongong, Wollongong, NSW 2522, Australia; bmeyer@uow.edu.au; 3 NUTRIM School of Nutrition and Translational Research in Metabolism, Maastricht University, 6200 MD Maastricht, The Netherlands

**Keywords:** recruitment, adherence, drop-out rates, omega-3 fatty acids, children, adolescents

## Abstract

Introduction: The influence of *n*-3 long-chain polyunsaturated fatty acids (*n*-3 LCPUFA) supplementation on health outcomes has been studied extensively with randomized controlled trials (RCT). In many research fields, difficulties with recruitment, adherence and high drop-out rates have been reported. However, what is unknown is how common these problems are in *n*-3 LCPUFA supplementation studies in children and adolescents. Therefore, this paper will review *n*-3 LCPUFA supplementation studies in children and adolescents with regard to recruitment, adherence and drop-out rates. Methods: The Web of Science, PubMed and Ovid databases were searched for papers reporting on RCT supplementing children and adolescents (2–18 years) with a form of *n*-3 LCPUFA (or placebo) for at least four weeks. As a proxy for abiding to CONSORT guidelines, we noted whether manuscripts provided a flow-chart and provided dates defining the period of recruitment and follow-up. Results: Ninety manuscripts (reporting on 75 studies) met the inclusion criteria. The majority of the studies did not abide by the CONSORT guidelines: 55% did not provide a flow-chart, while 70% did not provide dates. The majority of studies provided minimal details about the recruitment process. Only 25 of the 75 studies reported an adherence rate which was on average 85%. Sixty-five of the 75 studies included drop-out rates which were on average 17%. Conclusion: Less than half of the included studies abided by the CONSORT guidelines (45% included a flow chart, while 30% reported dates). Problems with recruitment and drop-out seem to be common in *n*-3 LCPUFA supplementation trials in children and adolescents. However, reporting about recruitment, adherence and dropout rates was very heterogeneous and minimal in the included studies. Some techniques to improve recruitment, adherence and dropout rates were identified from the literature, however these techniques may need to be tailored to *n*-3 LCPUFA supplementation studies in children and adolescents.

## 1. Introduction

Fatty acids, and especially the omega-3 long-chain polyunsaturated fatty acids (*n*-3 LCPUFA), are being researched extensively for a wide array of health outcomes varying from, but not exclusive to, cardiovascular diseases, depression and cognition [1,2,3]. As in every health related field, randomized controlled supplementation trials are the gold standard to demonstrate efficacy of *n*-3 LCPUFA [4]. For these trials, voluntary participants are needed, however recruitment of participants can be challenging, especially when it involves research in children and adolescents (<18 years) [5]. It has been reported that less than 31% of British studies funded by two funding bodies between 1994 and 2002 achieved their original recruitment target number [6]. Similarly, others have reported that up to 60% of the randomized controlled trials (RCT) fail to meet their participant target or need an extension [7] and this percentage might be even higher in paediatric and adolescent studies [8,9]. However, even after the recruitment phase, difficulties with conducting research do not end, because drop-out and non-adherence are also common. Drop-out in RCT is normal and attrition rates can vary enormously from 0 up to 65% [10,11,12]. Compliance and adherence are often used interchangeably, but are not the exactly same. Compliance is the extent to which the behaviour of a person coincides with the advice given by a doctor or researcher. The term compliance has received criticism because of its paternalistic connotation [13] and because it implies patient passivity [14]. As a more neutral term, adherence has been suggested, which presumes that the person agrees with the advice given by a doctor or researcher [14]. We choose to use the term adherence in the current manuscript. Adherence issues, are common, with non-adherence ranging anywhere from 3.5 to 80% [15,16]. One must also be aware that there is no one single definition of adherence. This means that somebody who is considered non-adherent in one study, might be considered adherent in another (e.g., one study defined a participant as non-adherent when the participant took less than 75% of the prescribed medicine or supplements, while another used a cut-off level of <80%). As low recruitment rates, high drop-out and high non-adherence are common and have serious consequences [6,17,18], it is important to study factors which possibly affect recruitment, drop-out, and adherence rates.

In 2013, we started a one-year long double blind randomized *n*-3 LCPUFA supplementation trial in healthy Dutch adolescents called Food2Learn [19]. We experienced difficulties in the recruitment, drop-out and adherence of the study participants. Furthermore, many other n3- LCPUFA supplementation studies have had the same difficulties (personal communication). However, a review of recruitment, adherence and drop-out rates in nutrition interventions and in specific *n*-3 LCPUFA supplementation studies in children and adolescents does not, to our knowledge, exist. Therefore, the aim is to execute a thorough review to summarize *n*-3 LCPUFA supplementation studies in children (2–12 years) and adolescents (12–18 years) with regard to recruitment effort, drop-out and adherence rates.

## 2. Materials and Methods

The Web of Science, PubMed and Ovid databases were searched up to 2 March 2017. We searched for human clinical trials including children aged between 2 and 18 years. We used the search terms: “Omega-3”, “DHA”, “EPA”, “LCPUFA” and “PUFA” in combination with “RCT”, “randomized controlled trial”, “supplementation”, “trial” or “fish oil” and “child*)“, “adolescent”, “school”, “preschool” or “toddler”. Furthermore, a myriad of reviews were checked for additional studies [20,21,22,23,24,25,26,27,28,29,30,31,32,33,34,35,36,37,38,39,40] and reference lists of all articles were hand checked for additional references. Moreover, a search of the Cochrane library was also conducted to identify reviews regarding *n*-3 LCPUFA supplementation. The studies included in the Cochrane reviews were checked for inclusion in the current study [41,42,43,44,45,46,47,48,49,50,51,52,53,54,55,56]. Lastly, for all included articles, the “cited by” option of Web of Science was checked (this option gives all articles that cite that specific article).

Studies were eligible for inclusion if they met the following criteria: (1) participants were aged between 2 and 18 years; (2) the study was a randomized placebo controlled *n*-3 LCPUFA supplementation trial; (3) the trial had at least 10 participants per treatment arm; (4) supplementation duration was at least 4 weeks; and (5) studies were published in English.

All papers were scanned by the first author, and the following information was extracted and entered in a database:

Participants’ characteristics: Age range of participants, percentage of girls, healthy participants or those with a diagnosed disease, and country in which the study was executed;

Study characteristics: Number of participants, number of measurement moments (i.e., how often did participants have to come to the research facility/how often did they have to fill out questionnaires), number of measurements, treatment condition, placebo condition, form of supplementation, if capsules were used then how many, if supplementation was taken under supervision, if supplement was taken in multiple dosages or once a day, whether an incentive was provided, duration of the study, manner in which adherence was assessed, adherence rate, whether fatty acids were determined in blood, and percentage of people who quit the treatment (hereafter called drop-out); and

Recruitment characteristics: Invited/responded or screened, started, finished as well as method of recruitment, recruitment setting, and study period.

The recruitment characteristics were defined as follows:

Invited: The total number of potential participants invited to participate;
Responded: The total number of potential participants who responded to the invitation or the number of participants that were screened for participation in the study; and
Started: The number of participants who were assessed as eligible and began supplementation.

Furthermore, efficiency percentages were calculated, namely: started/invited (dividing the number of people who started by the number of people who were invited times 100), started/responded (dividing the number of people who started by the number of people who responded times 100) and started/finished (dividing the number of people who finished by the number of people who started times 100).

As a proxy for adherence to the CONSORT guidelines, we noted whether the article included a flow-chart and whether the article provided the dates defining the period of recruitment and follow-up.

### Statistics

All extracted data were entered in SPSS (IBM SPPS statistics for Windows, version 24, Armonk, NY, USA). SPSS was used to calculate averages and SDs for the participant’ characteristics, study characteristics and recruitment characteristics. For comparison reasons outpatient clinics and hospitals were combined into one setting, which was named “hospital setting”. Countries were furthermore grouped in regions for comparisons (Europe, USA/Canada, Asia, Middle East, Australia, Africa and South America). When a study mentioned multiple compliance rates, these were combined into one compliance rate for the whole study.

## 3. Results

### 3.1. Study Characteristics

**Figure 1:**
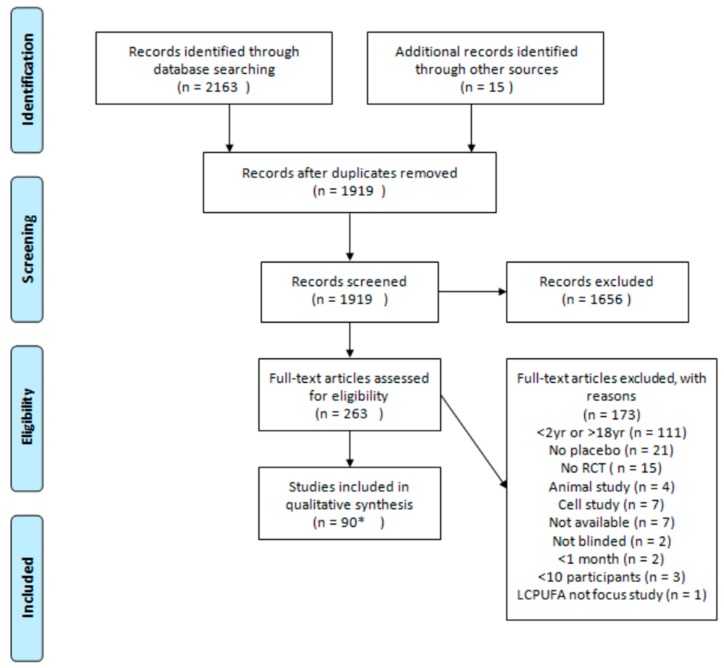
Flow diagram of study selection: 90 manuscripts were found reporting on 75 studies.

The original search led to 2163 hits. Upon first screening, 1656 articles were excluded, additional screening of the whole articles led to a further exclusion of 173 articles. Additional checking of the reference lists of reviews, included articles and forward checking led to 15 more studies being included (see Figure 1, adapted from [57]). Thus, in total, 90 papers, describing 75 studies, were included in this review. The characteristics of these studies can be found in Table 1. Fifteen studies focussed on healthy children. The other 60 studies focused on children with a disorder or disease, with attention deficit hyperactivity disorder (ADHD) being the most studied disorder (*n* = 21) (see Table 1). The majority of studies focussed on children (defined as aged between 2 and 12 years, *n* = 38) or both children and adolescents (*n* = 31). A minority of studies focussed only on adolescents (*n* = 6) (see Table 1). Duration of study varied from 4 to 52 weeks, with the majority of studies lasting 26 weeks or less (*n* = 59, 79%, see Table 2. Number of measurement moments (i.e., how often did participants have to come to the research facility/how often did they have to fill out questionnaires) varied from 2 to 16 with a mean of 3.7 (SD 2.7), the number of different measurements per moment varied from 1 to 19 with a mean of 4.9 (SD 3.7).

Two studies were published before the start of the CONSORT guidelines. Of the 73 studies that were published after the start of the CONSORT guidelines, 33 studies (45%) provided a flow diagram and 22 studies (30%) reported the dates defining the period of recruitment and follow-up.

### 3.2. Recruitment

Most of the studies included in this review did not report the number of children or adolescents that were invited to participate in the study, as only 11 out of 75 studies mentioned the number of participants that were invited. The total number of people invited to participate varied from 46 to 3562 (Mean (M) = 804.5, SD = 1083.28). The percentage of invited participants that eventually started the study ranged from 2.4 to 87% (see Table 3).

Forty out of 75 studies mentioned the number of participants that responded to the invitation or were screened for the study and this varied from 30 to 1556, with 12 to 100% of these people actually starting the study.

Most studies did not specify the exact method(s) of recruitment, mostly just mentioning the recruitment setting. Most studies recruited their participants from a hospital or outpatient clinic setting (*n* = 33). Other settings from which participants were recruited were schools (*n* = 23) and the community (*n* = 15). Nine studies reported multiple settings for recruitment; one study recruited participants from a summer camp for children with ADHD and other disorders; one study recruited from an online registry; one study recruited participants from those who participated in earlier studies; and eight studies did not mention the recruitment setting.

Looking at the efficiency percentages for started/invited, started/responded or started/finished for studies including those with an illness (averages 43.5%, 63.5%, and 83.1%, respectively) and those without (averages 36.2%, 53.1%, and 84.2%, respectively), there was a clear difference for started/invited and started/responded but not for started/finished. A comparison for average rates between studies including only children (38.8%, 62.2%, and 85.4%, respectively), only adolescents (15.5%, 56.4%, and 87.8%, respectively) or both (53.4%, 52.7%, and 79.6%, respectively) showed notable differences. For different recruitment settings, there were mainly clear differences for started/invited. However, for all rates, the school setting had the highest average rate: hospital (17.7%, 56.8%, and 82.9%, respectively), community (29.9%, 57.1%, and 81.7%, respectively), and school (46.0%, 64.6%, and 86.8%, respectively). Lastly, when looking at these average efficiency percentages for the different continents, we also saw clear differences: Europe (35.3%, 64.3%, and 87.8%, respectively), North America (6.6%, 48%, and 77.7%, respectively), Asia (49.5%, 48.7%, and 92.6%, respectively), Africa (NA, 28%, and 91.1%, respectively), Middle East (33.2%, 64.6%, and 79%, respectively), Australia (72.9%, 69.5%, and 70.7%, respectively), and South America (54%, 93.2%, and 90.9%, respectively).

### 3.3. Supplementation

Most studies used capsules as the form of supplementation (*n* = 57), however there were also some other approaches (see Table 2). The number of capsules that participants were instructed to take also varied widely from 1 to 12 capsules a day, with some studies basing the dose per body weight of the participant (see Table 2). Moreover, a huge range of different placebos was used (see Table 2).

### 3.4. Adherence

The included studies mentioned a wide variety of methods to measure adherence: capsule count (or product weighting) (*n* = 30), diaries or tick-off forms (*n* = 13), interviews face to face/via phone/ via e-mail (*n* = 11), taking the capsules under supervision (*n* = 8), and blood values (*n* = 5) (see Table 4). Thirteen studies used more than one method to assess adherence. Furthermore, 23 studies did not specify how or whether they assessed adherence. The way in which adherence was reported in the studies also varied greatly. Some studies mentioned percentages of capsules taken, the average number of capsules taken per day, blood values, or just mentioned that adherence was good or mentioned how many students were excluded due to non- adherence.

Twenty-five studies mentioned a specific percentage of adherence, which varied from 60 to 97%, mean 85% (SD 10.1). In addition, the levels of capsules that needed to be taken to be considered as being adherent differed per study, varying from 65 to 90%. Other studies defined adherence as the number of days of not taking capsules.

Looking at the adherence percentage between studies in healthy and diseased children, there seemed to be a slightly lower average adherence in diseased children (M = 83.7%, SD = 11.9), compared to healthy children (M = 87.6%, SD = 7.1). When we looked at the different age groups recruited, there seemed to be a lower average adherence in the child only group (M = 82.5%, SD = 9.5), compared to adolescents (M = 89.2%, SD = 1.1) or the combined group (M = 88.5%, SD = 11.2). The difference in average adherence in different recruitment settings was less clear; hospital (M = 86%, SD = 10.6), community (M = 89.5%, SD = 7.8), and school (M = 83.9%, SD = 11.6). The average adherence rate also differed between continents with a lower average rate in Australia and USA/Canada: Europe (M = 87.5%, SD = 9.3), USA/Canada (M = 78.7%, SD = 6.5), Asia (M = 92%, SD = 1.4), Africa (M = 95%, SD = 0.5), Australia (M = 79.7%, SD = 13.5), and South America (M = 94.5%, just one study). There seemed to be a tendency for higher average adherence when capsules were used (M = 88.2%, SD = 8.0) instead of food (M = 74.8%, SD = 14.3) or drinks (M = 81.5%, SD = 9.2), or other forms of supplementation (M = 80.3%, SD = 18.0). Some studies mentioned that participants took capsules under supervision, but they did not show a higher mean adherence (M = 82.8%, SD = 15.2) than those that did not have supervision of capsule intake (M = 86.2%, SD = 8.3). Seven studies, that reported adherence, reported that participants consumed capsules more than once a day while 12 studies, that reported adherence, mentioned that the capsules were only taken once a day. There was no difference in average adherence between those two methods of supplementation (M = 87.3%, SD = 9.4 vs. M = 87.6%, SD = 7.8). Fifteen studies, reporting adherence, mentioned talking to parents or participants either via telephone or face to face (or via e-mail) during the study about the supplementation to increase adherence [61,67,69,70,72,90,91,107,111,113,131,134,135,136,143]. The studies that included a phone call did not have a higher average adherence rate (M = 81.5%, SD = 9.5) than those that did not include a phone call (M = 86.2%, SD = 10.3). There were three studies that provided some form of incentive [98,107,146], however only one of these studies reported an adherence percentage. Forty-six studies mentioned that they took either blood or cheek samples, but only five studies mentioned that they used blood as an adherence measure [61,85,86,117,134].

### 3.5. Drop-Out

Sixty-five of the 75 included studies mentioned a drop-out rate or included numbers which made it possible to calculate the drop-out rate. The average drop-out was 17% (SD 13%), but it varied between 0% and 58% (see Table 4). There was no clear difference in average drop-out rate between studies in healthy (mean = 16.5%, SD = 11.5) and diseased populations (M = 17.9%, SD = 13.7). There was a difference in average drop-out with regard to the recruited age group: children M = 15%, SD = 11.1), adolescents (M = 12.3%, SD = 7.3) or both (M = 21.5%, SD = 14.9); with a higher average drop-out rate in the combined age group.

There was also no clear difference in mean drop-out between recruitment setting: hospital (M = 15%, SD = 11.1), community (M = 18.2%, SD = 8.6) or school (M = 15.3%, SD = 13). Differences could be seen in the average drop-out according to the continent on which the study was executed: Europe (M = 13.3%, SD = 9.8), USA/Canada (M = 23.1%, SD = 11.6), Asia (M = 6.8%, SD = 7.4), Africa (M = 11.6%, SD = 3.9), Middle East (M = 20.1%, SD = 16.7), Australia (M = 34.9%, SD = 7.8), and South-America (M = 9.1%, just one study). When looking at different forms of supplementation, no clear differences in average drop-out rate could be seen: capsules (M = 17.5%, SD = 12.9), food (M = 14.1%, SD = 14.9), drinks (M = 19%, SD = 13.7), and others (M = 17.9%, SD = 8.4). Eight studies who reported drop-out rate mentioned that capsules were taken under supervision, this seemed to lead to somewhat lower average drop-out rate (M = 13%, SD = 15.6), compared to the 57 studies in which participants did not take the capsules under supervision (M = 17.9%, SD = 15.6). Sixteen studies that reported drop-out rate divided the capsules over multiple intake moments (M = 17.2%, SD = 9.3). This did not seem to increase or decrease the average drop-out rate if compared to those studies that specified one intake moment (M = 17.1%, SD = 13.6). Fourteen studies that noted drop-out rate reported that they contacted the participants during the study. Studies that did so seemed to have a slightly higher average drop-out rate (M = 20.4%, SD = 11.4) than studies that did not contact participants during the study (M = 16.5%, SD = 14.4). Of the studies that reported giving participants an incentive, two mentioned a drop-out rate, this was on average 15.3% (SD 20.4). Studies that did not state an incentive had an average drop-out rate of 17.4% (SD 12.7). Of the 65 studies that mentioned a drop-out rate, 50 specified a reason for drop-out (six did not have drop-out, and nine did not specify the drop-out). Fifty-two different reasons for drop-out were mentioned, with the most common reasons mentioned being lost to follow-up, poor or no compliance or inability to take supplement.

## 4. Discussion

We conducted a thorough review to examine recruitment, adherence and drop-out rates in *n*-3 LCPUFA supplementation studies in children and adolescents, in order to identify strategies which can be implemented to improve those rates. Even though the CONSORT guidelines clearly state what data need to be included in the report of a RCT, the majority of the included studies did not provide a flow-chart (55% did not) or the dates defining the period of recruitment and follow-up (70% did not).

### 4.1. Recruitment

The majority of studies provided minimal details about the recruitment process. The low number of studies that reported the number of participants that they invited and screened is, however, not uncommon in research studies as similar numbers were reported by Toerien et al. who studied 129 studies in six major journals [148]. The literature does give some suggestions for methods that could increase recruitment; for example, telephone calls to those who do not reply, an opt-out system (participants contact the researchers if they do not want to participate, please do note that this is not legal in all countries), including incentives, making trials open, and in person recruitment [149]. The use of clinical referral is also suggested to be related to higher recruitment rates, as most patients will have a trusting relationship with their doctor [150]. When we looked at the research setting (hospital, community, school), though, the mean started/invited rate and mean started/responded rate seemed to be slightly higher in the school setting. However, in the studies that looked at diseased populations, the average percentage efficiency of started/invited and started/responded was higher than studies looking at healthy populations (M = 43.5% vs. M = 36.2% and M = 63.5% vs. M = 53.1%, respectively).

It has been shown that in adolescents, giving monetary incentives does improve response rates and has a positive effect on their willingness to participate in studies [151]. However, the provision of monetary incentives might be considered unethical in children/adolescents [152,153]. One might thus consider a form on non-monetary incentive, for example in Food2Learn participants received a cinema voucher [19]. In the current review, there were only three studies that provided an incentive and these studies did not have remarkably higher recruitment rates. Hence, more studies that do provide incentives are needed to elucidate whether or not incentives improve recruitment. Moreover, there are myriad reasons as to why somebody would or would not participate in a study. There are participant characteristics which in adults have been associated with a higher chance of non-participation such as younger age, being male, lower social economic status, and lower education level [154,155]. However, in the limited number of studies on recruitment in children, no association between age or sex has been seen, although the education level of parents was associated with higher enrolment rates [5].

Beliefs about the effectiveness of the treatment may also play a role. Examples of reasons as to why adolescents did not participate informally given in Food2learn included: (1) the belief that *n*-3 LCPUFA are not effective in improving health; (2) the belief that they already consume sufficient amount of *n*-3 LCPUFA/already eat healthy; (3) the belief that participation will take too much time/effort; and (4) lack of interest in research in general. These factors should be taken into account during the research process and it seems wise to include explanations that most people do not get enough *n*-3 LCPUFA in their diet as well as elaborating on the possible health benefits of *n*-3 LCPUFA specific to the age group being assessed.

### 4.2. Adherence

Just 25 studies mentioned a specific adherence percentage, which varied between 60% and 97% with a mean of 85%. Moreover, most studies included in the current review used indirect adherence assessment methods (i.e., diaries, interviews, and capsules counts) which are all subject to problems with reporting bias and errors or intentional manipulation [156]. More direct methods such as the determination of fatty acid levels in the blood seems to be the most reliable method to assess adherence, which was done in only five studies. However, it should be noted that taking blood samples in younger children might not be acceptable for all parents or ethical committees and could therefore lead to lower recruitment numbers.

In the current review, there was no difference in mean adherence in those studies where participants received a telephone call to try and increase adherence compared to those in which participants received no telephone call (M = 81.5% vs. M = 87.7%). There were only three studies that provided an incentive and only one of these studies provided an adherence percentage, which was 75%. There seemed to be a higher average adherence of capsules (M = 88.2%) compared to other forms of supplementation (M = 74.8%, M = 81.5%, M = 80.3%, for food, drink and other forms, respectively). Lastly, there was no difference in the mean adherence between those who took capsules multiple times a day compared to those who took capsules only once a day (M = 87.3% vs. M = 87.6%). It is however important to remember that all these findings are based on only 25 studies that mentioned an adherence rate.

Other studies suggest factors that are associated with higher adherence in children and adolescents, these include: sociodemographic factors (i.e., older children and older adolescents are less likely to be adherent, and boys are less likely to be adherent), disease associated factors (i.e., if the disease also has positive symptoms the person is less likely to be adherent), the belief and attitude that a person has towards the treatment (i.e., those that belief that the treatment will be effective are more likely to be adherent), their mood (i.e., those with depression are less likely to be adherent) and the social context (i.e., those who are supported by family and friends are more likely to be adherent) [157,158]. Methods to increase adherence rates have also been suggested. Methods that have been employed to increase adherence include: educating participants about adherence, making medicine (or supplementation) more palatable, providing incentives/tokens, and involving parents or schools [159,160]. However, one must take into consideration that the vast majority of studies looking at which methods can help increase adherence have been executed in a medical setting with patients requiring medications and these results do not by definition translate to nutritional interventions in healthy participants or those with diagnosed disorders such as ADHD.

Some suggestions for improving adherence for *n*-3 LCPUFA supplementation studies may include: providing sufficient information about the importance of adherence (i.e., explaining the importance of adherence to get valid results), getting parents involved, and providing appropriate incentives [159,160].

### 4.3. Drop-Out

In the current review, the average drop-out was 17% (range 0–58%). Three studies mentioned some form of incentive [98,107,146] and they reported a slightly lower average drop-out than those that did not use (or did not report) an incentive (M = 15.3% vs. M = 17.4%). There were differences in average drop-out rates between continents, with drop-out rates being higher in Australia (M = 34.9%), USA/Canada (M = 23.1%), and the Middle East (M = 20.1%) compared to Europe (M = 13.3%), Africa (M = 11.6%) and Asia (M = 6.8%). We can only speculate about explanations for this difference (e.g., individualistic vs. collective societies) and do point out that these differences have to be interpreted with caution as the number of studies per continent did differ greatly. A number of methods to decrease drop-out in studies involving adults has been suggested. They include emphasizing the benefit of participation, flexible scheduling of appointments, regular positive communication from the research team to the participants (e.g., birthday and Christmas cards, newsletters, etc.), a consistent research staff so participants can build a bond with the researchers, and appropriate incentives [150,161,162,163]. Other strategies that have been suggested include decreasing the complexity of the treatment and limiting the number of follow-up visits to the bare minimum [164]. Furthermore, a combination of multiple strategies is suggested to be most effective in increasing retention [161,164]. All these methods to decrease drop-out have been studied in adults; more research on methods to decrease drop-out in children and adolescents in RCT is warranted.

Suggestions for decreasing drop-out in *n*-3 LCPUFA supplementation trial include: keeping in regular contact with the participants, providing flexible appointment possibilities, providing incentives for participants and providing reminders. With regard to the supplement, one should keep the regime as simple as possible e.g., one (concentrated) capsule per day [150,161,162,163,164].

### 4.4. Strengths and Limitations

Limitations of the current review include the fact that many of the included studies did not report all data on recruitment, dropout and (assessment of) adherence. Due to the incomplete reporting of data, results should be viewed with caution. The main advantage of the current review is the fact that we included all studies investigating *n*-3 LCPUFA supplementation in children/adolescents regardless of whether they were healthy children/adolescents or children/adolescents with a disease or disorder.

## 5. Conclusions

The conclusions drawn are based on minimal reporting from the included studies in this review. Less than half of the included studies abided by the CONSORT guidelines. Problems with recruitment and drop-out seem to be common in *n*-3 LCPUFA supplementation trials in children and adolescents. However, since the reporting about recruitment, adherence and dropout rates was very heterogeneous and minimal in the included studies, we cannot provide specific suggestions to improve LCPUFA supplementation studies in children and adolescents.

## 6. Recommendations

It is important for future studies to report on recruitment effort and rate, adherence (including the method of assessing adherence) and drop-out rates according to the CONSORT Guidelines.

Suggestions from other scientific areas to increase recruitment, adherence and minimize drop-out include: the provision of sufficient information about the importance of adherence (i.e., explaining the importance of adherence to get valid results), getting parents involved, provision of appropriate incentives, emphasizing the benefit of participation, being flexible with the scheduling of appointments, the research team engaging in regular positive communication with the participants, having a consistent research staff member so participants can build a bond with the researchers and to keep the supplementation regime as simple as possible.

## Figures and Tables

**Table 1:** Characteristics of studies.

Reference	Age Range or Mean (SD)	Gender (%Female)	Population: Healthy, Disorder or Disease	Country
[58]	3–15	44	Acute lymphoblastic leukemia	Egypt
[59]	6–12	25	ADHD	Iran
[60]	7–15	NR	ADHD	Iran
[61]	11–12	31	ADHD	Canada
[62]	8–14	0	ADHD	The Netherlands
[63]	6–16	41 (after intervention)	ADHD	Israel
[64]	7–12	20	ADHD	Sweden
[65]	6–12	38	ADHD	Iran
[66]	6–12	NR	ADHD	Japan
[67,68]	8–18	15	ADHD	Sweden
[69]	12–16	0	ADHD	UK
[70,71]	6–13	23	ADHD	Australia
[72]	6–12	27	ADHD	Sri Lanka
[73]	7–13	41	ADHD	Israel
[74,75]	7–12	23	ADHD	Australia
[76]	6–13	13	ADHD	USA
[77]	8–13	25	ADHD	Israel
[78]	6–12	22	ADHD	USA
[79]	6–12	22	ADHD	Germany
[80,81]	6–13	34	ADHD	Israel
[82]	7–12	43 (after intervention)	ADHD or lower IQ	China
[83]	6–14	15	ADHD	Australia
[84]	6.9–11.9	NR	ADHD	Canada
[85]	8–16	48	Aggressive behaviour	Mauritius
[86]	6–14	42	Asthma	USA
[87]	8–12	56	Asthma	Australia
[88]	10–12	31	Asthma	Taiwan
[89]	10.2 (2.5) fish oil, 11.9 (3.1) control	48	Bronchial asthma	Japan
[90]	3–8	11	Autism	USA
[91]	5–8	NR	Autism	USA
[92]	2–5	26	Autism	Canada
[93]	3–10	17	Autism	USA
[94]	6–17	48% placebo, 46% flax oil	Bipolar disorder	USA
[95]	7.3–9.5	54	CF	Italy
[96]	5–16	47	Crohn’s disease	Italy
[97]	5–12	33	DCD	UK
[98]	10.6	43	Dyslexia	Finland
[99]	15–18	100	Dysmenorrhea	USA
[100]	4–12	NR	Epilepsy	Egypt
[101]	7–9	53	Healthy	South-Africa
[102]	8–14	50	Healthy	Indonesia
[103]	9–12	51	Healthy	Japan
[104]	9–10	50	Healthy	Sweden
[105]	10–12	49	Healthy	UK
[106]	8–10	52	Healthy	UK
[107]	5–7	NR	Healthy	Canada
[108]	8–10	0	Healthy	USA
[109]	6–10	46	Healthy	Australia and Indonesia
[110]	3–13	46	Healthy	Australia
[111]	8–14	51	Healthy	Spain
[112]	4	47	Healthy	USA
[113]	10–12	100	Healthy	Turkey
[114]	13–16	50	Healthy	UK
[115]	9–12	47	Healthy	Thailand
[116]	8–13	47	Hyperlipidaemia	Italy
[117]	14 (2)	31	Hypertriglyceridemia and low LDL	USA
[118,119,120,121]	6–11	49	Iron deficiency	South-Africa
[122]	8–12	15	Literacy problems	UK
[123]	8–12	58	Malnourished	Mexico
[124]	5–14	56	Migraine	Iran
[125]	7–14	NR	MDD	USA
[126]	6–12	NR	MDD	Israel
[127]	10–18	59 (after intervention)	Metabolic syndrome	Iran
[128]	9–17	47	NAFL and obesity	Turkey
[129,130]	11–15	14	NAFL and overweight	Poland
[131,132,133]	6–16	58	NAFL	Italy
[134]	10.8 (2.8)	48	NAFL and overweight	Italy
[135]	8–18	0	NAFL	Canada
[136,137]	14–17	56	Obesity	Sweden
[138,139]	13–15	0	Overweight	Denmark
[140]	9–18	NR	Overweight + insulin resistance	Mexico
[141]	5–10	NR	PKU	Italy
[142]	6–18	18	Tourette’s Disorder	USA
[143,144,145]	10 (7)	45	Type-1 type-1 hyperphenylalaninemia, HPA	Italy
[146,147]	6–10	47	Underperforming	UK

ADHD = attention deficit hyperactivity disorder; NAFL = non-alcoholic fatty liver; MDD = major depressive disorder; DCD = Developmental Co-ordination Disorder; CF = cystic fibrosis, NR = not reported.

**Table 2:** Treatment characteristics per study.

Reference	Treatment per Day Unless Otherwise Stated	Placebo	Form of Supplementation	Number of Capsules	Duration ^b^ (Weeks)
Healthy					
[108]	DHASCO ^a^: 400 or 1200 mg DHA	Corn oil	Capsules	6	8
[106]	800 mg FO: 400 mg DHA, 56 mg EPA	Olive oil	Chewable capsules	2	16
[113]	670 mg FO	Olive oil	Capsules	2	16
[110]	2400 mg FO and 600 mg evening primrose oil: 174 mg DHA, 558 mg EPA, 60 mg GLA.	Palm oil	Capsules	6	28.6
[104]	174 mg DHA, 558 mg EPA, 60 mg GLA	Palm oil	Capsules	6	12 + 12 (open)
[102]	1260 mg DHA rich oil: 652 mg DHA, 101 mg EPA	Placebo oil (656 mg LA, 87 mg ALA)	Capsules	6	12
[101]	Fish flour: 892 mg of DHA per week	Placebo spread contained bread flour	Margarine	NA	14.9
[107]	14–21 mg DHA, 20–30 mg AA	Placebo supplement	Sachets to mix into foods	2–3 sachets	30
[103]	FO: 3600 mg DHA, 840 mg EPA per week	50% soybean oil, 50% rapeseed oil (4200 mg LA per week)	Bread and sausages	NA	12
[114]	541 mg FO: 116 mg DHA, 165 mg EPA	Sunflower oil	Capsules	2	12
[112]	DHASCO-S ^a^: 400 mg DHA	High oleic sunflower oil	Capsules	2	16
[115]	FO: 1 g DHA, 200 mg EPA	Soybean oil	Chocolate milk	NA	15.6
[109]	88 mg DHA, 22 mg EPA	Unclear	Drink	NA	52
[105]	500 mg DHASCO-S ^a^: 200 mg DHA, 4 mg EPA	Vegetable oil (15 mg ALA, 250 mg LA)	Capsules	5	8
[111]	FO in dairy drink 120 mg DHA, 60 mg EPA	Whole milk	Milk drink	NA	20
[117]	4 g FO: 1.5 g DHA, 1.86 g EPA	Corn oil	Unclear	Unclear	8 + 8 with 4 weeks wash-out in between
[100]	3 mL dose of 1200 mg FO: 240 mg DHA, 360 mg EPA.	Corn oil	Liquid oil	NA	12
[88]	FO: 125 mg DHA, 230 mg EPA	Corn oil	Capsules	Dependent on bw	16
[96]	3 g O3FA	Olive oil	Capsules	Dependent on bw	52
[92]	1.875 mL FO: 0.75 g of DHA + EPA. If well tolerated dose ×2 after 2 weeks.	Olive oil and medium chain triglycerides.	Liquid oil	NA	24
[65]	165 mg DHA, 635 mg EPA, 100 mg other O3FA	Olive oil	Capsules	NS	8
[67,68]	174 mg DHA, 558 mg EPA, 60 mg GLA	Olive oil	Capsules	6	12 + 12 (open)
[97]	FO and EPO: 174 mg DHA, 558 mg EPA, 60 mg GLA	Olive oil	Capsules	6	26
[79]	120 mg DHA, 600 mg EPA	Olive oil	Capsules	2	16
[122]	480 mg DHA, 186 mg EPA, 96 mg GLA, 864 mg LA, 42 mg AA, 8 mg thyme oil	Olive oil	Capsules	NR	12
[66]	DHA-rich fish oil: 3600 mg DHA 700 mg EPA per week.	Olive oil	Milk and bread	NA	12
[76]	480 mg DHA, 80 mg EPA, 40 mg AA, 96 mg GLA	Olive oil	Capsules	8	16
[143,144,145]	LCPUFA supplementation: varying dosage	Olive oil	Capsules	1 per 4 kg of bw	52
[94]	Flax seed oil: 0.55 to 6.6 g ALA	Olive oil	Capsules	Varying up to 12	16
[89]	FO: DHA 7.3 ± 11.5 mg/kg of bw, EPA 17.0 ± 26.8 mg/kg of bw	Olive oil	Capsules	Dependent on bw: 6–12	43.6
[83]	PCSO-524 ^c^: 16.5–22 mg DHA, 21.9–29.2 mg EPA	Olive oil, lecithin and coconut oil	Capsules	Dependent on bw: 3–4	14
[86]	Drink containing FO (1.6 g DHA, 3 g EPA) and borage oil (3.0 g GLA)	Control drink with high oleic safflower oil	Drink	NA	12
[70]	EPA-rich FO: 108 mg DHA, 1,109 mg EPA or DHA-rich FO: 1,032 mg DHA, 264 mg EPA	Safflower oil	Capsules	4	16 + 16 + 16
[90]	FO: 1.1 g DHA + EPA	Safflower oil	Pudding packet	2 pudding packs	12
[123]	FO: 180 mg DHA, 270 mg EPA	Soybean oil	Capsules	3	12
[135]	2 g FO: 1200 mg DHA + EPA	Sunflower oil	Capsules	4	24
[87]	FO: 1.2 g O3FA	Sunflower oil	Capsules, salad dressing and margarine	4	24
[72]	FO and EPO oil: 592.74 mg O3FA	Sunflower oil	Capsules	2	26
[58]	1 g FO:120 mg DHA, 180 mg EPA	Sunflower oil	Capsules	Unclear	24
[61]	100–400 mg DHA, 500–100 mg EPA	Sunflower oil	Capsules	Dependent on bw: 2–4	16
[129,130]	AO: 450–1300 mg O3FA (DHA: EPA in 3:2 proportion)	Sunflower oil	Capsules	Dependent on bw	24
[116]	AO: 500 mg DHA or FO:500 mg DHA + EPA	Wheat gern oil	Capsules	1	16
[131,132,133]	AO: 250 or 500 mg DHA	Germ oil	Capsules	1	26.1
[134]	AO: 250 mg DHA	Germ oil	Capsules	NR	26
[95]	Algae triacylglycerol 100 mg DHA/kg/day 1st month then 1 g DHA/day	Germ oil	Capsules	4	52
[93]	AO: 200 mg DHA	Corn oil + soy bean oil	Capsules	1	26
[146,147]	AO: 600 mg DHA	Corn oil + soy oil	Capsules	3	16
[138,139]	4.9 g FO: 892 mg DHA, 191 mg EPA	6:1:1 mix of palm shortening, soy oil, and rapeseed oil	Bread	NA	16
[141]	2.5–4 g FO (12% DHA, 18% EPA)	Blackcurrant seed oil (45.7% LA, 18% GLA, 14% ALA)	Capsules	Dependent on bw: 5-8	26
[62]	650 mg DHA, 650 mg EPA	Normal margarine (1 g LA)	Margarine	NA	16
[99]	FO: 720 mg DHA, 1080 mg EPA	1800mg lactose	Capsules	2	8+8
[125]	200 mg DHA, 1400 mg EPA, 400 mg other O3FA	Placebo capsule	Capsules	2	12
[136,137]	FO and EPO: 290 mg DHA, 930 mg EPA, 100 mg GLA	Placebo	Capsules	10	12 + 12 with 6 weeks wash-out in between
[91]	FO: 1.1 g DHA + EPA	Identical placebo	Pudding packet	2 pudding packs	6
[128]	1000 mg PUFA	Placebo	Capsule	1	52
[63]	2 g sage oil: 1 g ALA	Lactose placebo	Capsules	2	8
[60]	240 mg DHA, 360 mg EPA	Placebo	Capsules	2	8
[64]	FO: 2.7 mg DHA, 500 mg EPA	Placebo	Capsules	1	15
[127]	2.4 g omega-3	Vitamin E or placebo	Tablets	NR	8
[84]	100 mg DHA, 250 mg EPA, 25 mg phospholipids	Sunflower oil	Capsules	According to bw: 1–2	16
[124]	1 g FO: 120 mg DHA, 180 mg EPA	Placebo capsule	Capsules	1	At least 8 weeks
[78]	Algae oil: 345 mg DHA	Placebo capsule	Capsules	1	16
[59]	241 mg DHA, 33 mg EPA, and 180 mg omega-6	Identical placebo	Capsules	1	10
[118,119,120,121]	FO: 155 mg DHA, 29 mg EPA	Placebo	Capsules	2	15
[142]	Varying 500–6000 mg O3FA	Placebo	Capsules	Varying up to 12	20
[140]	Salmon oil: 360 mg DHA, 540 mg EPA	Placebo (corn starch, lactose, magnesium stearate and polyvinyl pyrrolidone)	Capsules	NR	4
[85]	300 mg DHA, 200 mg EPA, 400 mg ALA, 100 mg of DPA	Drink without omega-3	Drink	NA	24
[77]	FO: 96 mg DHA, 153 mg EPA or *n*-3 LC-PUFA containing PLs: 95 mg DHA, 156 mg EPA	Rapeseed oil	Chocolate flavoured spread	NA	13.1
[73]	240 mg LA, 60 mg ALA, 95 mg mineral oil	Vitamine C capsules	Capsules	1	7
[69]	FO and EPO: 174 mg DHA, 558 mg EPA, 60 mg LA.	Medium chain triglycerides	Capsules	6	12
[126]	200 mg DHA 400 mg EPA, or 180 mg DHA, 380 mg EPA	Olive oil or safflower oil	Capsules	1–2	16
[74,75]	FO and EPO: 174 mg DHA, 558 mg EPA, 60 mg GLA	Palm oil	Capsules	6	30
[98]	500 mg ethyl-EPA	Triglycerides and cellulose	Capsules	NR	12.9
[80,81]	1–15 weeks: 120 mg EPA + DHA 16–30 weeks: 60 mg EPA + DHA	Cellulose	Capsules	4	15 + 15
[82]	321 mg DHA, 42.2 g EPA per 100 g egg	Ordinary egg	Egg	1	13.1

^a^ DHASCO is an algal triglyceride DHA; ^b^ Some studies gave duration in months or number of days supplementation was received, we recalculated the duration to weeks; ^c^ PCSO-524 is an lipid extract of the New Zealand green-lipped mussel; bw: body weight, NA: not appropriate, NR: not reported.

**Table 3:** Recruitment effort and recruitment rates.

Reference	Invited	Responded/Screened	Started	Finished	Started/Invited %	Started/Responded %	Started/Finished %	Recruitment Method	Recruitment Setting	Study Period
[141]	NS	NS	21	21	-	-	100	NS	Department of Paediatrics	NS
[66]	46	40	40	40	87	100	100	Parents of summer camp participants were asked.	Summer camp for children with psychiatric disorders	NS
[98]	107	107	61	61	57		100	Teachers nominated children with reading difficulties	School	Autumn 2005–January 2006
[131,132,133]	NS	NS	60	60	-	-	100	NS	Hospital	NS
[115]	NS	NS	180	180	-	-	100	NS	School	NS
[116]	NS	NS	36	36	-	-	100	NS	Hospital	8 month period
[146,147]	1376	675	362	359	26	54	99	Parents of underperforming children received a letter inviting their children to take part in the formal screening assessments.	School	NS
[105]	NS	NS	90	88	-	-	98	Via advertising in newspapers and schools	Community and schools	NS
[88]	NS	298	197	192	-	66	98	Participants with asthma diagnosis were recruited from elementary schools through parent conferences	Schools	NS
[82]	1556	1556	179	171	12	12	96	Children were screened from students in two township primary schools	Schools	NS
[62]	NS	372	79	76	-	21	96	Via hospital and advertising at schools.	Hospital and schools	NS
[72]	NS	422	98	94	-	23	96	NS	Outpatient treatment program	NS
[114]	NS	408	196	189	-	48	96	NS	School	NS
[58]	NS	100	70	65	-	70	93	NS	Hospital	NS
[117]	NS	NS	42	39	-	-	93	NS	Hospital	NS
[118,119,120,121]	NS	926	321	294	-	35	92	Parents were invited to an information meeting.	School	November 2009–November 2010
[127]	NS	NS	90	83	-	-	92	NS	Cardiovascular Research Centre	NS
[60]	NS	NS	75	69	-	-	92	NS	Outpatient ADHD clinic	2007
[103]	NS	230	179	166	-	78	92	Via advertisements	Community	NS
[85]	NS	938	200	184	-	21	92	Via parents who themselves had participated in a study.	Participants earlier study	November 2009–December 2011
[123]	NS	59	55	50	-	93	91	Parents were invited to a meeting at which the study procedures were explained and a written informed consent from the tutors and a verbal assent from their children were obtained.	School	NS
[95]	NS	NS	41	37	-	-	90	NS	Hospital	NS
[101]	NS	NS	183	164	-	-	90	NS	School	NS
[138,139]	3652	NS	87	78	2	-	90	Subjects were recruited via addresses obtained from the Danish Civilian Person Register.	Community	NS
[111]	NS	NS	119	107	-	-	90	NS	School	NS
[99]	NS	NS	42	37	-	-	88	NS	School	NS
[134]	NS	118	58	51	-	49	88	NS	Hospital	May 2012–September 2014
[113]	NS	44	33	29	-	75	88	Via public flyers	Community	NS
[135]	NS	30	24	21	-	80	88	NS	Hospital	NS
[87]	NS	NS	45	39	-	-	87	NS	NS	Over period of 16 mo.
[108]	NS	48	38	33	-	79	87	NS	NS	NS
[112]	NS	405	202	175	-	50	87	NS	NS	NS
[86]	NS	NS	43	37	-	-	86	NS	Outpatient clinic	NS
[65]	NS	NS	120	103	-	-	86	NS	Hospital	NS
[97]	189	129	117	100	62	91	86	Letters of invitation were sent to parents of children who were identified by teachers.	School	NS
[78]	NS	250	63	54	-	25	86	Via advertisements	Community	NS
[79]	NS	334	110	95	-	33	86	Via health professionals, teachers, leaflets handed out to support groups, leaflet distributed at community centres and advertisements in a free of charge regional newspaper.	Community, Health professionals, schools, support groups.	NS
[64]	NS	NS	109	92	-	-	84	NS	Hospital and secondary treatment centres	January 2005–June 2007.
[129,130]	NS	86	76	64	-	88	84	NS	Hospital	2008-2011
[92]	NS	101	38	32	-	38	84	NS	Hospital	December 2010–December 2013
[107]	NS	NS	37	31	-	-	84	NS	NS	NS
[143,144,145]	NS	NS	24	20	-	-	83	NS	NS	Recruited over 6 month
[125]	NS	178	23	19	-	13	83	Via advertisements and clinician referrals.	Community and referral	July 2011–May 2014
[109]	NS	932	780	643	-	84	82	Via advertisement at schools and media advertisement.	Schools	Auguet 2003–April 2005
[136,137]	108	47	31	25	29	66	81	NS.	Outpatient clinic	NS
[73]	NS	~300	78	63	-	26	81	Via advertisement on radio health program, in health newspapers and in ADHD clinics.	Community and ADHD clinic	January 2007–June 2007
[80,81]	NS	247	200	162	-	81	81	Advertisements in newspapers, on the Internet and medical centres.	Community	NS
[91]	863	118	57	45	7	48	79	E-mail invitations to in registry and longitudinal study of families of children affected by ASD.	Online registry	18 September 2012–31 December 2012
[67,68]	NS	NS	75	59	-	-	79	NS	Hospital	October 2004–August 2006
[128]	NS	NS	138	108	-	-	78	NS	Outpatient clinic	March 2010–June 2012
[122]	NS	NS	41	32	-	-	78	NS	School	NS
[106]	NS	511	450	348	-	88	77	Via school	Schools	NS
[89]	NS	NS	30	23	-	-	77	NS	Hospital	January 1994–March 1995
[142]	NS	NS	33	25	-	-	76	Via community, hospital and through patient association.	Community and referral	NS
[69]	NS	138	76	57	-	55	75	School and parent group circulated screening information to all potential eligible families	Schools and parent groups	NS
[83]	NS	351	144	108	-	41	75	NS	NS	NS
[77]	250	102	83	60	33	81	72	Newspaper advertisement	Community	July 2004–January 2005
[126]	NS	NS	28	20	-	-	71	NS	Hospital	NS
[93]	NS	143	48	34	-	34	71	Via recruitment flyers across campus and sent to autism support groups.	Campus, autism support groups	NS
[61]	NS	NS	37	26	-	-	70	NS	ADHD clinic	NS
[90]	NS	32	27	19	-	84	70	NS	Outpatient autism clinic	5 November 2008–25 June 2009
[84]	NR	NR	37	26	-	-	70	NS	NS	NS
[104]	NS	162	154	105	-	95	68	Via teachers who informed families	School	December 2009–July 2011
[76]	NS	193	50	33	-	26	66	NS	Community	NS
[74,75]	NS	201	167	109	-	83	65	NS	NS	Start March–May 2004
[70]	NS	199	96	57	-	48	59	Via media releases, television interviews, newspaper advertisements, school newsletters, and flyers.	Community and School	June 2007–June 2009
[110]	560	447	408	227	73	91	56	Via information sessions and school newsletters.	Schools	December 2010–May 2011
[94]	NS	NS	51	24	-	-	47	NS	Hospital	November 2001–March 2005
[63]	NS	NS	40	17	-	-	43	NS	ADHD clinic	NS
[59]	NS	NS	40	NS	-	-	-	NS	Outpatient ADHD clinic	June 2009–March 2010
[124]	NS	NS	25	NR	-	-	-	NS	Hospital	NS
[140]	142	NS	76	NS	54	-	-	From previous sample children with insulin resistance were identified and invited	Community	NS
[102]	NS	NS	233	NS	-	-	-	Via school	School	NS
[100]	NS	NS	70	NS	-	-	-	NS	Hospital	NS
[96]	NS	NS	38	NS	-	-	-	NS	Hospital	NS

NS = not specified.

**Table 4:** Adherence and drop out characteristics per study.

Reference	Adherence Assessment	Adherence Mean or nr. of Part. Non-Adherent	Blood FA Determined?	Drop-Out Rate (%)	
				Treatment	Placebo
**Healthy**					
[114]	Supervision and tick-off form	Active: 88.4%, Placebo: 88.5%	Y	3.1	6.1
[115]	Supervision	NR	Y	0	0
[108]	NR	NR	Y	Low DHA: 20; High DHA: 7.1	17
[112]	Capsule count	Nearly 100%	Y	7.1	5.6
[101]	Supervision	Active: 94.8%, Placebo: 94.5%	Y	11	9.8
[106]	Pill diary by teacher or parent	Active: 68.4%, Placebo: 66.7%	Y	24	21
[102]	NR	NR	Y	NR	NR
[103]	NR	>90%.	Y	6.7	7.8
[109]	Sachet count and diary (Australia)/Supervision (Indonesia)	Australia: 73-84%	Y	27	34
		Indonesia: 85-87%		3.6	5.3
[111]	Interview	small increase in DHA in supplemented group	Y	NR	NR
[107]	Diary	*n* = 6	Y	NR	NR
[105]	Parental signing of diary card	>80%.	N	NR	NR
[113]	NR	NR	N	5.9	19
[110]	Supervision	Phase 1: 59%, Phase 2: 61%	N	47	42
**With disorder or illness**					
[141]	NR	NR	Y	0	0
[140]	Pill count	Active: 93%, Placebo: 96%	Y	NR	NR
[82]	Supervision	count of consumed eggs showed good compliance and % of adherence to treatment was 100%	Y	5.6	3.2
[127]	Pill count	pill count revealed no essential irregularities	Y	13.3	Vit. E: 0, Placebo: 10
[84]	NR	NR	NR	NR	NR
[80,81]	Pill count	*n* = 14	N	20	18
[83]	Pill count, compliance diary and telephone call	96.7%	N	23	23
[95]	NR	*n* = 2 DHA supplementation induced a median plasma DHA enrichment of 5% suggesting adherence	Y	14	5
[138,139]	Interview	90%	Y	NR	NR
[79]	Capsule count	*n* = 1	Y	13	11
[86]	Diary and blood values	80–85%	Y	21.1	11.1
[77]	Phone calls and product weighting	*n* = 6	Y	Phospholips: 38, Fish oil: 25	24
[118,119,120,121]	Supervision	95.4%	Y	6.9	9.9
[61]	Blood	NR	Y	CO	CO
[94]	Capsule count and diary	>75%	Y	42	64
[70]	Capsule count	EPA: 83%, DHA: 86% , LA: 85%	Y	CO	CO
[87]	Capsule count	75%	Y	NR	NR
[76]	Diary	88%	Y	28	40
[143,144,145]	NR	NR	Y	17	17
[131,132,133]	Capsule count and interview	excellent in all groups	Y	NR	NR
[62]	Product weighting	*n* = 1	Y	0	5.1
[135]	Capsule count and interview	NR	Y	0	25
[78]	Capsule count	Active: 96.7%, Placebo: 100%	Y	15	13
[93]	Capsule count	excellent	Y	21	38
[136,137]	Capsule count	*n* = 1	Y	CO	CO
[90]	Parent interview	Active: 69%, Placebo: 75%	Y	36	23
[69]	Capsule count	FA changed in the expected direction.	Y	24	30
[89]	NR	NR	Y	27	14
[64]	Capsule count	NR	Y	30	19
[116]	Capsule count	DHA: 96.5%, DHA + EPA: 96.9%, Placebo: 96.7%	Y	DHA: 0, DHA + EPA: 0	0
[117]	Blood value	NR	Y	CO	CO
[129,130]	Capsule count	95.5%	Y	21	11
[128]	Capsule count	NR	Y	NR	NR
[134]	Blood values	*n* = 5	Y	14	10
[98]	NR	According to parents children took the capsules carefully	Y	NR	NR
[88]	Supervision and capsule count	Pill count: 91%	Y	0	0
[92]	NR	there was no overlap between the distributions of plasma levels between groups at week 24	Y	21	11
[65]	Capsule count	*n* = 5	N	NR	NR
[97]	Capsule count and diary	Period 1: 88.7%, Period 2: 85.5%	N	17	12
[91]	Parents e-mail	Active: 69%, Placebo: 83%	N	28	14
[85]	Parent interview and blood values	Average number of drink per week 6.5.	N	10	22
[73]	Capsule count	Active 7.88 capsules left; Placebo: 14 capsules left	N	18	21
[125]	NR	89–97%	N	10	23
[142]	NR	NR	N	18	31
[67,68]	Parent interview	Period 1: 93.4%, Period 2: 93.3%	N	CO	CO
[126]	NR	*n* = 5	N	NR	NR
[122]	Capsule count	Active: 90.4%, placebo 86.6%	N	23	21
[74,75]	Capsule count and diary	*n* = 2	N	CO	CO
[63]	Capsule count	NR	N	60	55
[59]	NR	NR	N	NR	NR
[124]	NR	NR	N	NR	NR
[100]	NR	NR	N	NR	NR
[96]	NR	Compliance was optimal	N	NR	NR
[66]	NR	NR	N	0	0
[146,147]	Diary	At school: 75%	N	0.6	1.1
[72]	NR	NR	N	2	6.1
[58]	NR	*n* = 5	N	8.6	5.7
[60]	NR	NR	N	NR	NR
[123]	Diary and capsule count	NR	N	0	20
[104]	Interview	Active: 94%, Placebo: 92%, Period 2: 91%	N	CO	CO
[99]	NR	*n* = 1	N	CO	CO

CO: cross-over study, NR: not reported.
